# How negative anthropomorphic message framing and nostalgia enhance pro-environmental behaviors during the COVID-19 pandemic in China: An SEM-NCA approach

**DOI:** 10.3389/fpsyg.2022.977381

**Published:** 2022-08-22

**Authors:** Shuai Zhou, Yibo Wang

**Affiliations:** ^1^School of Economics and Management, Zhoukou Normal University, Zhoukou, China; ^2^Asia Europe Institute, University of Malaya, Kuala Lumpur, Malaysia

**Keywords:** pro-environmental behavior, environmental responsibility, environmental empathy, perceived threat, perceived vulnerability, nostalgia, SOR model, protection motivation theory

## Abstract

Although extensive research has been conducted on promoting pro-environmental behaviors among consumers, little is known about whether and how negative anthropomorphic message framing (NAMF) and nostalgia affect pro-environmental behavior. To provide a framework for explaining pro-environmental behavior, this study integrates protection motivation theory, the stimulus-organism-response model, and message framing. To create the model of the influences on pro-environmental behavior, NAMF was employed as the external stimulus; the sense of environmental responsibility, environmental empathy, perceived threat, and perceived vulnerability as the psychological and cognitive response factors; pro-environmental behavior as the final decision of consumers; and nostalgia as the moderating variable. An online questionnaire was distributed and 380 usable questionnaires were collected using convenience sampling and analyzed using two complementary approaches: partial least squares structural equation modeling (PLS-SEM) and necessary condition analysis (NCA). PLS-SEM results showed that pro-environmental behavior was significantly affected by NAMF (β = 0.313, *t*-value = 5.583), environmental responsibility (β = 0.207, *t*-value = 3.994), and perceived threats (β = 0.252, *t*-value = 4.889). Meanwhile, an increase in nostalgia increased the effect of NAMF and environmental responsibility on pro-environmental behavior. The NCA results revealed that NAMF (*d* = 0.108, *p* < 0.001) and perceived threat (*d* = 0.209, *p* < 0.001) were key factors of pro-environmental behavior. In addition, for high level of pro-environmental behavior (>80%), NAMF (12.1%) and perceived threat (39.6%) are required. Finally, we offer several suggestions based on the results of our empirical research. For example, marketing and service offerings should be tailored to the needs of masses with different nostalgic tendencies to enhance their pro-environmental behaviors.

## Introduction

As social transformation accelerates and consumerism becomes more prevalent, environmental concerns have become more prominent (Panda et al., 2020; Busby, 2021). An increasing number of urban residents are realizing that worsening environmental issues are connected to their own lives and their behavior contributes to urban environmental problems (do Paço et al., 2013; Nekmahmud and Fekete-Farkas, 2020; Patak et al., 2021). A recent study indicated that unreasonable consumption habits and consumption patterns contribute significantly to environmental degradation (Sheng et al., 2019). A consumer behavior strategy that encourages consumers to abandon the consumption style of wastefulness and actively practice green consumption that respects nature and pursues health is a practical necessity for promoting sustainable economic and social development and a pressing theoretical issue in consumer behavior. Therefore, to solve urban environmental problems, a correct understanding of the pro-environmental behavior of urban residents is required (Lindenberg and Steg, 2007; Zhang et al., 2020; Xi and Wang, 2022), as well as providing urban residents full credit for their contribution to environmental protection. In general, pro-environmental behavior is an action that is taken by humans to protect the environment and prevent its degradation (Stern, 2000).

Scholars have investigated the mechanisms of pro-environmental behavior based on different theoretical foundations and diverse fields. Among them, the most representative theories are the theory of planned behavior (TPB) (De Groot and Steg, 2007; De Leeuw et al., 2015; Carfora et al., 2017; Yang et al., 2020; Alzubaidi et al., 2021) and the norm-activation model (Onwezen et al., 2013; Han et al., 2015; van der Werff and Steg, 2015; Gao et al., 2022). Studies using these theories have demonstrated the importance of rational cognitive factors in the development of pro-environmental behaviors. As a result, rational cognitive factors, such as environmental concerns and subjective norms, have been continuously incorporated into the system of factors influencing pro-environmental behavior. Nevertheless, several empirical studies have indicated that logical cognitive elements have no appreciable influence on pro-environmental behavior (Steg, 2005; Nisbet et al., 2009; Miliute-Plepiene et al., 2016; Liang et al., 2019; Kao and Du, 2020). These findings question the role of rational cognitive factors in pro-environmental behavior, leading scholars to shift the focus from rational variables to the role and mechanisms of emotional factors.

Emotions are closely associated with consumer behavior (Westbrook, 1987; Arnold and Reynolds, 2009; Troiville et al., 2019). However, there have been few empirical studies on the relationship between emotions and pro-environmental actions (Vining, 1987; Bissing-Olson et al., 2016), especially in the context of COVID-19. COVID-19 brought the world to a standstill, which is a testament to the frailty of globalization (El-Said and Aziz, 2022). The lockdowns due to COVID-19 have been linked to psychological distress, such as loneliness, unhappiness, depression, and anxiety (Zhou et al., 2021). Those experiencing negative emotional states such as loneliness and depression are prone to nostalgia (Wildschut et al., 2006). Unfortunately, the link between nostalgia and pro-environmental behavior is not well understood (Zhang et al., 2021). Studies have shown that nostalgia can assist in making socially beneficial decisions and achieving social goals. Compared to non-nostalgic charity appeals, nostalgic appeals are more likely to increase charitable giving, volunteering, and helping behavior (Merchant et al., 2011; Zhou et al., 2012). However, it is not clear whether nostalgia influences people's pro-environmental behavior, which is a subset of pro-social behavior at different levels. Moreover, little is known from past research about the moderators of the relationship between natural anthropomorphism and pro-environmental outcomes (Williams et al., 2021a). To fill these research gaps, this study was the first, to our knowledge, to use nostalgia as a moderator in a framework investigating the factors influencing pro-environmental behavior.

**Figure 1:**
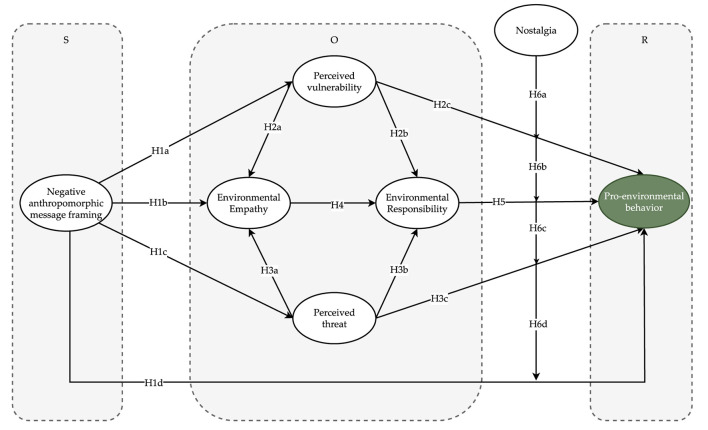
Theoretical framework.

Nevertheless, encouraging people to connect emotionally with nature is not easy. Scholars have begun to analyze how anthropomorphism could be applied to establish a positive emotional connection with the environment. However, as anthropomorphism is embodied in different forms, its impact on participation in environmental behavior also differs (White et al., 2011). Few studies have explored the relationship between anthropomorphism and pro-environmental behavior during the COVID-19 pandemic (Williams et al., 2021a), let alone different forms of anthropomorphism. Previous scholars have divided message frames into positive and negative information frames (White et al., 2011), which are conditional phenomena and rarely work in isolation (Karpinska-Krakowiak et al., 2020). Meanwhile, the process of pro-environmental behaviors is difficult to explain in studies based on the TPB because external stimulus factors are not considered (Yue et al., 2020). Based on the above analysis, an integrated model (Figure 1) based on stimulus-organism-response (SOR), protection motivation, and message frame theories was built to explain the mechanisms influencing the pro-environmental behavior of residents during the COVID-19 pandemic.

Protection motivation theory provides an alternative theoretical perspective for explaining environmental behavior (Bockarjova and Steg, 2014). It employs a broader range of predictor variables than the TPB, the norm activation model, and the value-belief-norm theory, enhancing our understanding of attitudes and behaviors driving pro-environmentalism, which can be used to encourage pro-environmental actions to mitigate environmental threats (Bockarjova and Steg, 2014). Mehrabian and Russell (1974) developed the SOR model of early psychology by introducing the concept of the “organism” to emphasize that the external environment influences individual behavior through changes in psychological perceptions. According to this model, various situational elements (stimuli) lead to cognitive and emotional psychological changes in the recipient (organism), which in turn facilitate behavioral responses. The message framework refers to the concept that the same message content, presented in different ways, will be understood differently by the recipient. Messages can be described in terms of negative or positive information frames. Negative information frames are descriptions of information that cause losses or negative effects to people. In our study, we combined negative framing and anthropomorphism as the external stimulus of SOR theory, which few studies have done before (Karpinska-Krakowiak et al., 2020), to investigate how the negative form of anthropomorpism affects pro-environmental behavior. In addition, we selected two core variables (perceived threat and perceived vulnerability) in protection motivation theory and environmental empathy and responsibility as consumers' cognitive and emotional responses to stimuli of negative framing and anthropomorphism. We then employed the variable of pro-environmental behavior as the final decision of the consumers. This is a pioneering study on the relationship between perceived threat, perceived vulnerability, environmental empathy, and environmental responsibility.

Overall, our work offers theoretical and applied insights. First, the more comprehensive model can provide better insight into the multifaceted motivations of individuals (Mehrabian and Russell, 1974). Second, numerous studies have examined the role of nostalgia in promoting prosocial behavior (Zhou et al., 2008, 2012; Routledge et al., 2011; Stephan et al., 2014; Juhl et al., 2020). Although pro-environmental behavior is a form of pro-social behavior (Ito and Li, 2019; Sun et al., 2021; Zelenski and Desrochers, 2021), scholars have not paid sufficient attention to the relationship between nostalgia and pro-environmental behavior (Zhang et al., 2021). To our knowledge, this is the first pro-environmental behavior study to consider nostalgia as a moderator variable in a research model. We investigated whether different levels of nostalgia affect consumer behavior regarding environmental issues. Third, this study investigates the underlying mechanisms and develops a comprehensive framework for understanding how negative anthropomorphic message framing (NAMF) influences pro-environmental behavior. Finally, most previous studies employed structural equation modeling (SEM; CB-SEM and partial least squares (PLS)-SEM), which is useful for elucidating the net effects of variables, but they do not address the complexity of causal relationships that exist asymmetrically in the real world (Tho, 2019). Therefore, PLS-SEM and necessary condition analysis (NCA) were both used in this study to identify and validate the variables' sufficient and necessary conditions.

## Theoretical background and hypotheses development

### Anthropomorphism, message frame, and pro-environmental behavior

Anthropomorphism refers to the application of human physical traits, motives, and emotional inclinations to non-human objects (Mota-Rojas et al., 2021). Anthropomorphism has a broad variety of marketing uses and studies have examined how anthropomorphism influences individuals' behavior. Anthropomorphism has a positive effect on customer preferences, engagement, and purchase intentions (Kim et al., 2016; Schroll et al., 2018). A study of anthropomorphism and its relationship to nature demonstrated a link between anthropomorphism and environmental concerns (Borovik and Pensini, 2022). Minimizing social distance and fostering social connections is critical for assisting people in engaging in pro-environmental behaviors (Schultz, 2002). Research has shown that anthropomorphizing nature can enhance people's sense of connection to it and encourage sustainabile behaviors (Tam, 2015b; Williams et al., 2021a,b). Thus, anthropomorphism may encourage prosocial behavior and increase a person's desire to purchase green products. Indeed, those who anthropomorphize nature are more likely to engage in social connection (Tam et al., 2013) and empathy (Tam, 2013b, 2015a) with the environment.

The message frame effect, also known as the ornamental effect, refers to the various ways in which the same information can be presented to elicit various interpretations by the information's receiver (Tversky and Kahneman, 1985). A message frame can be positive or negative. While message frames are crucial for consumer behavioral choices (Levin et al., 1988), no scholarly consensus exists over which information frameworks are more successful in enhancing pro-environmental behavior. Message framing is a conditional phenomenon and seldom operates in isolation (Olsen et al., 2014; Amatulli et al., 2019). Therefore, it is unclear whether anthropomorphism, when combined with negative message framing, impacts consumers' environmental behaviors and the possible mechanism of this impact. To address this, we used a multi-theoretical framework and two complementary approaches, PLS-SEM and NCA, to investigate the impact of NAMF on pro-environmental behavior.

### Hypotheses development

#### NAMF

Message frameworks are crucial for consumers' behavioral choices (Levin et al., 1988). Negative message frames are used to describe messages that result in losses or negative consequences for people (Krishnamurthy et al., 2001; Gerend and Cullen, 2008) and trigger the recipient's expected feelings of shame and guilt (Amatulli et al., 2019). A negative framing emphasizing negative environmental implications increased customer desire to utilize environmentally friendly biofuels more than access-based or hybrid-based frameworks (Moon et al., 2016). Anthropomorphism relates environmental things to humans, increasing the similarities between individuals and environmental topics, making the perceived psychological distance smaller (Chen et al., 2020). According to interpretive-level theory, a closer psychological distance tends to elicit a lower level of consumer interpretation and enables people to feel how nature is being violated (Zhang, 2014).

It is possible to trigger active behaviors by activating adaptive biases, including anthropomorphism and an innate tendency to respond to negative contexts (Haselton and Nettle, 2006; Haselton et al., 2015). Positive and negative messages increase the vividness of the information and elicit a stronger emotional response (Karpinska-Krakowiak et al., 2020; Kusmanoff et al., 2020). Negative target messages may be more effective than positive target messages because they generate negative emotions (Witte, 1992; Chang and Wu, 2015), such as fear, anger, and vulnerability, which are perceived as a threat by the individual. According to The Khoa et al. (2021), who developed a framework for negative anthropomorphic messages, consumers may be persuaded to maintain social distancing using anthropomorphic persuasion, such as by replacing the word coronavirus with “the deadly Mr. COVID-19” or showing terrifying images of the virus.

Anthropomorphism is intrinsically tied to human sociocognitive development (Epley et al., 2007). If people care about the environment, their emotions and perceptions must be aligned with human emotions and perceptions (Clayton et al., 2011). As a general rule, people are more likely to accept the behavior of others who are similar to themselves (Bandura, 2001) and may require some degree of anthropomorphism to achieve identification and empathy for non-human subjects (Tam et al., 2013; Prguda and Neumann, 2014). It has been demonstrated that when humans anthropomorphize nature, their sense of connection to it improves (Tam et al., 2013), generating a strong natural empathy (Tam, 2013b, 2015a) which increases conservation behavior (Tam et al., 2013; Liu et al., 2019). Therefore, we propose the following hypotheses:

H1a. Negative anthropomorphic message framing has a positive influence on perceived vulnerability.
H1b. Negative anthropomorphic message framing has a positive influence on environmental empathy.
H1c. Negative anthropomorphic message framing has a positive influence on perceived threats.
H1d. Negative anthropomorphic message framing has a positive influence on pro-environmental behavior.

#### Perceived vulnerability and perceived threats

Perceived vulnerability refers to how people feel in the face of adverse circumstances. Empathy refers to the ability of a person to feel, understand, and even share the feelings of others (Escalas and Stern, 2003; Marjanovic et al., 2012; Rohani et al., 2018). A behavioral immune system more sensitive to environmental threats or changes is found in more vulnerable individuals (Tam, 2013a). Consequently, these individuals feel compelled to invest more effort into protecting the environment to reduce their chances of exposure to environmental hazards (Jiang et al., 2021). As vulnerability is recognized as a condition of impairment, more responsibility must be extended to those who are especially vulnerable (Gross and McGoey, 2015). People who are aware of the vulnerability of the environment feel responsible for it and choose to make environmentally friendly purchases (Channa et al., 2022). Related research has found that people feel more responsible for helping those they perceive to be as vulnerable (Back and Lips, 1998; Chasteen and Madey, 2003; Moche and Västfjäll, 2021). When environmental subjects are anthropomorphized, people will treat the environment in the same way as they treat people, encouraging people to actively engage in pro-environmental behavior. Previous research has examined how farmers' adaptation decisions are influenced by psychological factors, such as perceived vulnerability and capacity (Wang Y. et al., 2019). They found that perceived vulnerability influenced Chinese farmers' intention to adopt environmental practices. We conclude that individuals feel a sense of environmental responsibility and urge to protect the earth when they see a crying planet.

Perceived threat was developed based on a social psychology study of fear. It is a significant component affecting environmental behavior and is related to the degree to which people perceive damage from the external environment. According to protective motivation theory, people confront risks using two cognitive processes: threat assessment and reaction evaluation. In reaction to dangers, people employ protective behaviors depending on their threat assessment. Danger intensity is viewed as a trigger for protective behavior and a belief about the threat's outcome, and individuals behave positively when they believe the threat is controllable and unmanageable. Due to the anthropomorphization of environmental issues, individuals may see the controllability of the environment and engage in pro-environmental acts. Using a large sample of 27 nations, Oreg and Katz-Gerro (2006) illustrated that environmental concerns, perceived threats, and behavioral restrictions may all impact willingness to sacrifice and a variety of pro-environmental behaviors. In particular, perceived threats might reinforce pro-environmental behavior when pro-environmental standards become dominant (Fritsche et al., 2010). Environmental problems are more likely to be perceived as significant by individuals with a higher perception of environmental threats than those with a lower perception, motivating them to take part in pro-environmental behaviors (O'Connor et al., 1999). In addition, concern for nature is linked to an individual's underlying values and belief systems (Stern et al., 1995), meaning that they develop a feeling of empathy for nature when they feel a threat due to environmental problems (Berenguer, 2010). In summary, we propose the following hypotheses:

H2a. Perceived vulnerability positively influences environmental empathy.
H2b. Perceived vulnerability positively influences environmental responsibility.
H2c. Perceived vulnerability positively influences pro-environmental behavior.
H3a. Perceived threat positively influences environmental empathy.
H3b. Perceived threat positively influences environmental responsibility.
H3c. Perceived threat positively influences pro-environmental behavior.

#### Environmental empathy

Empathy for nature is the emotional experience of comprehending and sharing the natural world. It encompasses induced and character empathy (Tam, 2013b; Tam et al., 2013). According to the TPB, a person's emotions are critical in anticipating their behavioral intentions (Ajzen, 1991). Emotional reactions to environmental concerns and problems are conceptualized as persistent sentiments toward nature, especially when triggered by environmental challenges and crises (Liu and Lin, 2015). Empathy for creatures impacted by environmental difficulties has been shown to generate significant environmental attitudes and behaviors (Schultz P., 2000). In addition, the model of altruistic emotions also implies that the more empathy one has for nature, the more probable it is that one would sense nature's wellbeing, which may result in moral worries regarding nature conservation behavior (Batson, 2014). In summary, empathy for distressed natural beings may inspire concern for both the individual and the natural world and instill a sense of environmental responsibility in consumers. Thus, we propose the following hypothesis:

H4. Environmental empathy has a positive influence on environmental Responsibility.

#### Environmental responsibility

Environmental responsibility refers to an individual's sense of obligation to take action to address environmental issues or avoid the degradation of environmental quality and is the primary factor determining environmental behavior (Yue et al., 2020). According to the norm activation and value-belief-norm theories, environmental behavior implementation is contingent on the activation of individual norms that result in a sense of responsibility to adopt environmental behavior (Stern, 2000; Nordlund and Garvill, 2003). Individuals perceive environmental responsibility as a response to moral responsibility, which plays a key role in amplifying intrinsic motivation and reinforcing their willingness to act. Research has shown that by incorporating environmental responsibility into theoretical models of planned behavior, environmental behavioral intentions can be predicted more accurately (Hines et al., 1987; Achchuthan et al., 2017; Rezaei et al., 2019). Thus, we propose the following hypothesis:

H5. Environmental responsibility has a positive influence on pro-environmental behavior.

#### Nostalgia

Nostalgia is a complex, positive, self-relevant emotion that occurs when people reminisce about their past (Sedikides et al., 2015). Human behavior is heavily influenced by emotion (Vitell et al., 2013) and nostalgia has become an increasingly important psychological resource that enhances the sense of meaning in existence and promotes prosocial behavior among individuals. Nostalgia is positively correlated with prosocial behaviors (Lasaleta et al., 2014; Stephan et al., 2014). In addition, participants who viewed nostalgic advertisements were more willing to make charitable donations than those who viewed non-nostalgic advertisements and were more likely to volunteer and donate to charitable organizations (Zhou et al., 2012). Pro-environmental behavior is an important expression of pro-social behavior (Ramus and Killmer, 2007). However, few studies have examined how nostalgia affects consumers' pro-environmental behaviors. One study used structural equation modeling to examine how nostalgia affects tourists' pro-environmental behavior through subjective attitudes, perceived behavioral control, subjective norms, and meaning in life (Wu et al., 2020). Two studies revealed that personal nostalgia increases the likelihood of retaining and reusing products to protect nature (Wang et al., 2020; Zhang et al., 2021). Thus, we propose the following hypotheses:

H6a. Nostalgia positively moderates the relationship between perceived vulnerability and pro-environmental behavior.
H6b. Nostalgia positively moderates the relationship between environmental responsibility and pro-environmental behavior.
H6c. Nostalgia positively moderates the relationship between perceived threats and pro-environmental behavior.
H6d. Nostalgia positively moderates the relationship between negative anthropomorphic message framing and pro-environmental behavior.

## Methodology

The hypotheses of this study were investigated using PLS-SEM and NCA, with PLS-SEM assessing the adequacy of the antecedent conditions and NCA and analyzing the necessity of the antecedent conditions (Farmaki et al., 2022; McLeay et al., 2022; Sharma et al., 2022a). In combination with NCA, PLS-SEM facilitates the assessment of a model's necessary logic (Dul, 2016; Richter et al., 2020). The combined research method of SEM and NCA may assist in establishing whether a determinant has an average effect and/or a necessary effect on the outcome, providing a thorough understanding of the causal relationships between variables (Lee and Jeong, 2021). In this study, individual indicator weights, along with measurement errors, were estimated using PLS-SEM and then utilized to calculate composite scores for the defined latent variables (Hair et al., 2017). Next, the scores from this process are utilized in the NCA (Richter et al., 2020).

PLS-SEM requires a relatively small sample size, does not require sample data to follow a normal distribution, and can handle both reflective and formative indicators, making it more suitable for exploratory studies (Hair et al., 2019). Moreover, it can handle more complex models (Chin, 2010), making it suitable for this study. Smart PLS 3.3.9 was employed for data analysis. In recent years, NCA has emerged as a method for analyzing the necessary conditions to facilitate a particular outcome and is used to complement more traditional regression methods that analyze sufficiency conditions (Dul et al., 2020). The presence or absence of antecedent conditions directly impacts an outcome and NCA allows for a quantitative analysis of the level of antecedent conditions required to reach an outcome variable (Dul, 2016). In our study, NCA techniques were used to complement the SEM approach, which is well suited to adequacy analysis and includes the analysis of effect size and bottleneck conditions.

### Survey development and data collection procedure

Before conducting formal research, an experiment was conducted to test the validity of our manipulation of the negative frame. An experiment was conducted with 60 students at a university in central China which displayed two anthropomorphic images (Appendix 5): a positive anthropomorphic image of a smiling Earth and a negative anthropomorphic image of a tearful Earth. Each student was asked to answer four questions of positive and negative frames: “Do you think the campaign of protecting the Earth has a positive (negative) meaning” and “Do you think participating in protecting the Earth initiative will benefit you in any way?” (1 = strongly disagree; 5 = strongly agree). The results showed that the Cronbach's α of the positive frame was 0.741 and 0.828 for the negative frame, indicating that the questionnaire is credible. In addition, after reversing the results of the negative anthropomorphic message, we found a significant difference between the negative and positive frames [M negative frame = 4.333, M positive frame = 2.283, F_(119)_ = 193.164, *p* < 0.001], demonstrating the success of our manipulation of the negative and positive frames. The subjects produced positive and negative emotions when they saw smiling earth and crying earth, respectively.

Next, to explore the research hypothesis, a survey was conducted for 1 week on WJX, an online survey platform that offers specialized and reliable services (Wang D. et al., 2019). We clarified the purpose of the study and emphasized the anonymity and confidentiality of the participants. In total, 412 electronic questionnaires were received and 32 responses were deleted after filtering for straight lining (Sharma et al., 2022b) and missing data. The demographic data of the respondents are presented in Table 1.

**Table 1:** Subjects' demographics.

**Measure**	**Item**	**Frequency**	**Percent**	**Cumulative percent**
Gender	Male	193	50.8	50.8
	Female	187	49.2	100
Age	Below 20	9	2.4	2.4
	21–30	175	46.1	48.4
	31–40	126	33.2	81.6
	41–50	36	9.5	91.1
	50 and over	34	8.9	100
Edu	Junior high school or below	19	5	5
	Senior high school	46	12.1	17.1
	Junior college or university	188	49.5	66.6
	Master's degree or PhD	127	33.4	100
Income/month (RMB)	Less than 3,500	188	49.5	49.5
	3,500–6,000	101	26.6	76.1
	6,001–8,000	52	13.7	89.7
	8,001–9,999	22	5.8	95.5
	Over 10,000	17	4.5	100
	Total	380	100	

### Measurement procedure

The variables in this study were assessed using a variety of items derived from existing scales in previous research. The items in this research were measured using a five-point Likert scale (1 = greatly inaccurate, 5 = highly accurate). The questionnaire consisted of three main sections. In the first part, we presented a picture of an anthropomorphic and weeping Earth (Appendix 5), which was the material used in the experiment before the formal study to evoke negative emotions among the participants. In the second part, respondents' demographic information was provided. Based on existing measurement scales validated in previous research, in the third part, we presented seven variables: NAMF (Aggarwal and McGill, 2007), perceived vulnerability (Workman et al., 2008; Shafiei and Maleksaeidi, 2020), perceived threat (Liang and Xue, 2009), environmental empathy (Tam, 2013b), environmental responsibility (Stone et al., 1995), pro-environmental behavior (Stern, 2000), and nostalgia (Hepper et al., 2012).

## Results

### Common method bias, social desirability bias, and non-response bias

To test the questionnaire's validity and quality, three types of bias were analyzed: common method bias, social desirability bias, and non-response bias. To reduce the impact of common method bias, the English scales derived from foreign literature were back-translated and repeatedly compared and corrected, thus reducing the possibility of errors caused by ambiguity in the language. In addition, Harman's single-factor analysis was performed and seven variables were used to ensure that the collected data did not have common method bias. In this study, a single factor was constructed by combining these seven factors. The results indicated that the newly formed factor explains 31.448% of the variation, which is less than the 40% requirement (Babin et al., 2016). Hence, the data collected did not indicate a concern regarding common method bias.

Social desirability bias and non-response bias are common problems in survey research using convenient sampling methods. We found no significant differences between data collected in the pre- and post-periods of this study for either demographic or latent variables, demonstrating that non-response bias was not a significant problem in our study (Armstrong and Overton, 1977). Regarding social desirability bias, first, the survey was completely anonymous so that respondents could express their innermost thoughts, and we made it clear at the outset that their personal information would not be compromised to avoid social desirability bias (Konrad and Linnehan, 1995; Wei, 2021). Second, according to previous research (Park et al., 2019), all scales in our study were derived from high-quality studies to mitigate the social desirability bias. Third, regarding pro-environmental behavior, previous studies have indicated that the social desirability bias does not play a significant role (Kaiser, 1998; Milfont, 2009). Finally, in light of the data description of the measurement items (Appendix 2), there was no significant deviation in the values of the variables, indicating that social desirability bias may not pose a problem in this study (Sethi and Sethi, 2009).

### Assessment of the measurement model

To determine the convergent and discriminant validity of the measurement model, we used conventional criteria. First, average variance extracted (AVE) scores (>0.5) and outer loadings (>0.708) were used to test convergent validity (Hair et al., 2013). The findings in Table 2 show that all items were above the acceptable value, suggesting that this study attained convergent validity. Second, according to Hair et al. (2021), Cronbach's alpha, composite reliability (CR), and rho_A were used to assess the reliability of the measures. The three values were all above 0.7, the accepted cutoff point, demonstrating the reliability of the scale (Table 2).

**Table 2:** Reliability and convergent validity.

**Construct**	**Item**	**Loading**	**VIF**	**Cronbach's alpha**	**rho_A**	**CR**	**AVE**
EE	EE1	0.884	2.345	0.874	0.900	0.913	0.726
	EE2	0.878	2.567				
	EE3	0.889	2.699				
	EE4	0.748	1.650				
ER	ER1	0.917	2.445	0.818	0.832	0.892	0.735
	ER2	0.829	1.772				
	ER3	0.822	1.800				
NAMF	NAMF1	0.865	2.003	0.793	0.816	0.866	0.618
	NAMF2	0.820	1.747				
	NAMF3	0.743	1.588				
	NAMF4	0.707	1.567				
NO	NO1	0.715	1.941	0.879	0.946	0.906	0.661
	NO2	0.753	2.255				
	NO3	0.899	3.012				
	NO4	0.881	2.462				
	NO5	0.802	1.926				
PEB	PEB1	0.827	2.142	0.882	0.885	0.911	0.630
	PEB2	0.823	2.445				
	PEB3	0.743	2.091				
	PEB4	0.804	2.169				
	PEB5	0.783	2.087				
	PEB6	0.780	1.925				
PT	PT1	0.879	1.927	0.807	0.813	0.886	0.722
	PT2	0.813	1.580				
	PT3	0.855	1.874				
PV	PV1	0.871	2.746	0.889	0.890	0.923	0.750
	PV2	0.860	2.703				
	PV3	0.864	2.698				
	PV4	0.867	2.719				

*NAMF, negative anthropomorphic message framing; PV, perceived vulnerability; PT, perceived threat; EE, environmental empathy; ER, environmental responsibility; PEB, pro-environmental behavior; NO, nostalgia*.

In addition, the Fornell-Larcker criterion (Fornell and Larcker, 1981) and the heterotrait-monotrait correlation ratios (Henseler et al., 2015) were used to test the discriminant validity of the model. It is essential to achieve discriminant validity of the construct because it differentiates constructs and measures distinct concepts (Fornell and Larcker, 1981). The Fornell-Larcker criteria are based on the standardized outer loadings of each construct and the correlation between each latent variable and all other constructs. The square root of the extracted average variance must be larger than the component correlation coefficient (Table 3). The HTMT refers to the ratio of correlations between variables to correlations within variables, and an HTMT value greater than HTMT 0.85 (Henseler et al., 2015), or HTMT 0.90 (Gold et al., 2001) indicates a problem with discriminant validity. As shown in Table 4, the HTMT test resulted in values that met the HTMT 0.85 and HTMT 0.90 requirements, showing that the measurement model was determined to have sufficient discriminant validity. The results of the multicollinearity indicator with the variance inflation factor (VIF) are presented in Table 2. VIF values of less than three were considered ideal (Sarstedt et al., 2019). Our fit validity test indicated that the SRMR was 0.063, which is less than the recommended maximum of 0.08 (Hair et al., 2021). This indicated a good fit for the model. We also used goodness of fit (GOF) to evaluate the model fit of our study (Tenenhaus et al., 2005). Quality assessment of the research framework was performed as follows.


$$\begin{array}{l} GOF=\sqrt{\bar{AVE}}\;x\;\sqrt{\bar{R^{2}}}=\sqrt{0.692\;x\;0.351}=0.493 \end{array}$$

**Table 3:** Fornell and larcker.

	**EE**	**ER**	**NAMF**	**NO**	**PEB**	**PT**	**PV**
EE	**0.852**						
ER	0.538	**0.857**					
NAMF	0.504	0.387	**0.786**				
NO	0.086	0.092	0.248	**0.813**			
PEB	0.356	0.468	0.614	0.274	**0.794**		
PT	0.545	0.460	0.578	0.116	0.573	**0.850**	
PV	0.382	0.455	0.382	−0.027	0.234	0.307	**0.866**

*The diagonal numbers (bold values) are the square root of each variable AVE*.

**Table 4:** HTMT.

	**EE**	**ER**	**NAMF**	**NO**	**PEB**	**PT**	**PV**
EE							
ER	0.617						
NAMF	0.592	0.472					
NO	0.098	0.123	0.248				
PEB	0.400	0.545	0.724	0.268			
PT	0.635	0.565	0.715	0.143	0.677		
PV	0.417	0.533	0.445	0.065	0.266	0.363	

Considering the above results, the GOF was 0.493, which satisfies the 0.40 cut-off requirements for a substantial impact size (Wetzels et al., 2009). Compared with recent studies that used SRMR (Petrescu-Mag et al., 2021, 2022) and GOF (Asghar et al., 2021; Kopaei et al., 2021; Rastegari Kopaei et al., 2021) values to test the model fit, the model fit measurements of both SRMR and GOF in our study showed that our model is appropriate.

### Assessment of the structural model

**Figure 2:**
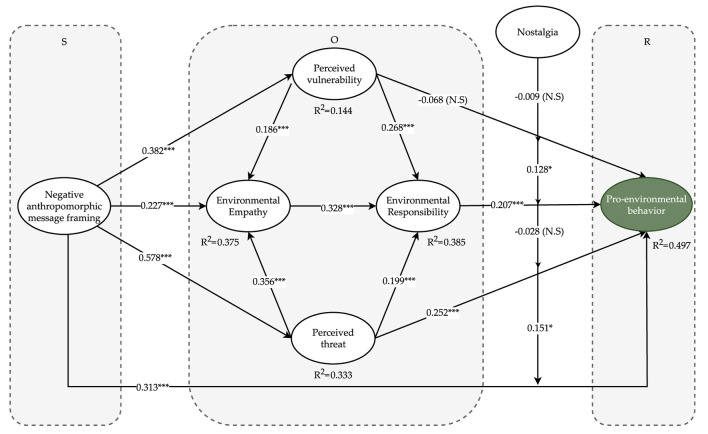
Inner model results. ***p-value < 0.001, **p-value < 0.01, and *p-value < 0.05.

Collinearity problems in the dataset may lead to distortion of the regression results. VIF was used to assess collinearity. To avoid collinearity problems, the VIF values should not exceed three (Hair et al., 2019). According to Table 5, the VIF values of all variables (1.187–1.728) fulfilled this criterion, indicating that collinearity was not a concern. In total of 5000 subsamples were utilized in a bootstrap technique to determine the significance of the route coefficients. The structural model is illustrated in Figure 2 and Table 5 shows that NAMF has a significant positive effect on perceived vulnerability (β = 0.382, *t*-value = 8.096), environmental empathy (β = 0.227, *t*-value = 4.287), perceived threat (β = 0.578, *t*-value = 15.961), and pro-environmental behavior (β = 0.313, *t*-value = 5.583). Thus, H1a, H1b, H1c, and H1d were supported. Perceived vulnerability was found to significantly increase environmental empathy (β = 0.186, *t*-value = 3.871) and environmental responsibility (β = 0.578, *t*-value = 15.961) but did not significantly affect pro-environmental behavior (β = −0.068, *t*-value = 1.575). Therefore, H2a and H2b are supported and H2c is rejected. We also found that perceived threat had a positive effect on environmental empathy (β = 0.356, *t*-value = 7.435), environmental responsibility (β = 0.199, *t*-value = 3.721), and pro-environmental behavior (β = 0.252, *t*-value = 4.889), supporting H3a, H3b, and H3c. Additionally, environmental empathy demonstrated a significant positive effect on environmental responsibility (β = 0.356, *t*-value = 7.435), while environmental responsibility also had a significant effect on pro-environmental behavior (β = 0.207, *t*-value = 3.994), thus supporting H4 and H5.

**Table 5:** Assessment of structural model.

**Hypothesis and path**	**Coefficiet**	**Std**	**T values**	***P* values**	**f^2^**	**VIF**	**Result**
ER -> PEB	0.207	0.052	3.994	^***^	0.073	1.484	Supported
NAMF -> PEB	0.313	0.056	5.583	^***^	0.165	1.728	Supported
NO -> PEB	0.116	0.037	3.152	0.002	0.030	1.089	Supported
PT -> PEB	0.252	0.052	4.889	^***^	0.083	1.669	Supported
PV -> PEB	−0.068	0.043	1.575	0.115	0.013	1.374	Not Supported
**PEB; R**^**2**^ **= 0.497; Q**^**2**^ **predict = 0.309**							
NAMF -> EE	0.227	0.053	4.287	^***^	0.052	1.615	Supported
PT -> EE	0.356	0.048	7.435	^***^	0.134	1.523	Supported
PV -> EE	0.186	0.048	3.871	^***^	0.047	1.187	Supported
**EE; R**^**2**^ **= 0.375; Q**^**2**^ **predict=0.266**							
EE -> ER	0.328	0.049	6.617	^***^	0.115	1.533	Supported
PT -> ER	0.199	0.053	3.721	^***^	0.045	1.446	Supported
PV -> ER	0.268	0.048	5.631	^***^	0.099	1.190	Supported
**ER; R**^**2**^ **= 0.385; Q**^**2**^ **predict = 0.280**							
NAMF -> PT	0.578	0.036	15.961	^***^	0.503		Supported
**PT; R**^**2**^ **= 0.333; Q**^**2**^ **predict = 0.237**							
NAMF -> PV	0.382	0.047	8.096	^***^	0.171		Supported
**PV; R**^**2**^ **= 0.144; Q**^**2**^ **predict = 0.106**							
PV*NO -> PEB	−0.009	0.044	0.212	0.832			Not Supported
NAMF*NO -> PEB	0.151	0.059	2.571	0.010			Supported
PT*NO -> PEB	−0.028	0.045	0.626	0.531			Not Supported
ER*NO -> PEB	0.128	0.053	2.427	0.015			Supported

* *p < 0.05*,

** *p < 0.01, and*

*** *p < 0.001*.

In addition, using the Smart-PLS algorithm function, structural model assessments were used to calculate the coefficient of determination (R^2^), which evaluates the explained variance. Four endogenous constructs were used: pro-environmental behavior, environmental empathy, environmental responsibility, perceived threat, and perceived vulnerability. The results in Table 5 reveal that the R^2^ values for the five endogenous constructs were 0.497, 0.375, 0.385, 0.333, and 0.144, respectively. Simultaneously, Stone-Geisser Q^2^ was calculated to assess the predictive value of endogenous variables. The findings in Table 5 show that the Q^2^ values for pro-environmental behavior (0.309), environmental empathy (0.266), environmental responsibility (0.280), perceived threat (0.237), and perceived vulnerability (0.106) were all above zero (Shmueli et al., 2016). To analyze the statistical power of our data, we calculated the effect size using f^2^. An f^2^ value less than or equal to 0.02 indicates a weak small relationship. If the value of the independent variable is larger than 0.15 and less than 0.35, then the relationship's moderate or medium strength may be assessed. If the independent variable had an effect greater than 0.35, the relationship was considered strong (Hair et al., 2019). As shown in Table 5, NAMF, perceived threat, and environmental empathy had the largest f^2^ values for pro-environmental behavior, environmental empathy, and environmental responsibility, respectively. The f^2^ values from NAMF to perceived threat and perceived vulnerability were 0.503, 0.171.

**Figure 3:**
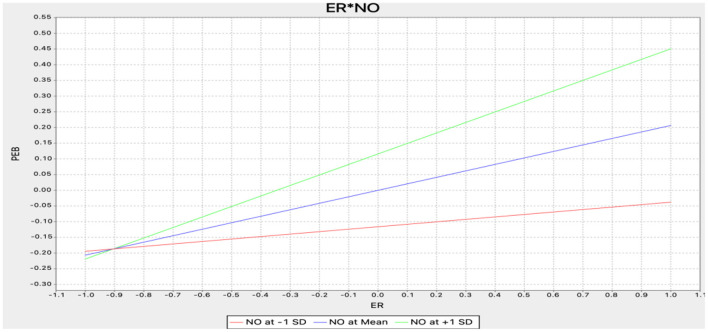
Moderating effect (ER*NO-PEB). ER, environmental responsibility; PEB, pro-environmental behavior; NO, nostalgia.

**Figure 4:**
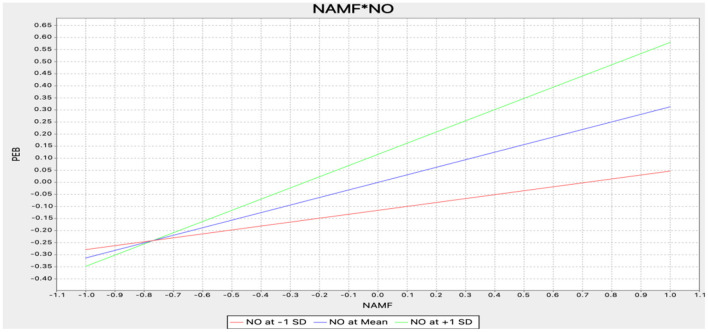
Moderating effect (NAMF*NO-PEB). NAMF, negative anthropomorphic message framing; PEB, pro-environmental behavior; NO, nostalgia.

An approach based on product indicators was used in Smart PLS to assess moderating effects (Henseler and Fassott, 2010). The results of the moderating effect tests are presented in Table 5. This study highlights the significant effects of the interaction between NAMF and nostalgia (NAMF^*^nostalgia) on pro-environmental behavior (β = 0.151, *t*-value = 2.571) and the interaction between environmental responsibility and nostalgia (environmental responsibility^*^nostalgia) on pro-environmental behavior (β = 0.128, *t*-value = 2.427), thereby supporting H6b and H6d. Figures 3, 4 show the positive moderating effects of NAMF and environmental responsibility on pro-environmental behavior. Unexpectedly, H6a and H6c were not supported.

### NCA result

**Figure 5:**
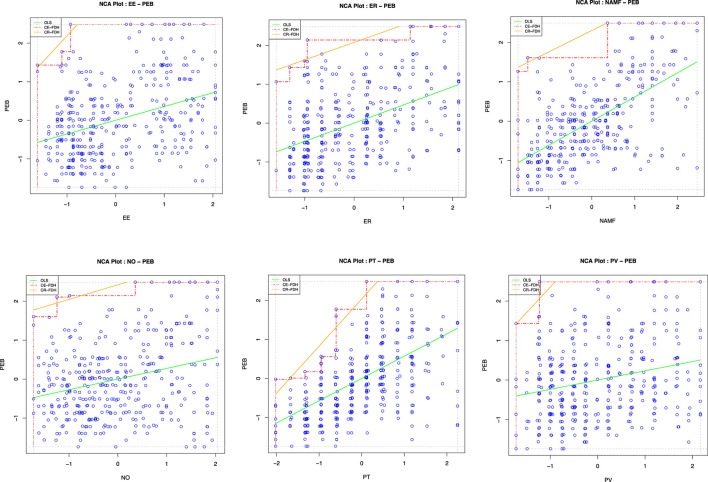
Scatter plots of necessary condition analysis.

NCA involves analyzing and interpreting necessary condition hypotheses using three components: a scatterplot, an effect size, and a bottleneck table (Toth et al., 2019). Instead of assessing average correlations, an NCA analysis displays regions in the scatter plots of dependent and independent variables, which might indicate the existence of a required condition (Dul, 2016; Richter et al., 2020). This study employed the NCA package in the R environment to perform NCA analysis (Dul, 2021). NCA was conducted using the latent variable scores obtained by PLS-SEM as a starting point (Richter et al., 2020). We attempted to determine whether NAMF, environmental empathy, environmental responsibility, nostalgia, perceived threat, and perceived vulnerability were necessary conditions for pro-environmental behavior. Figure 5 shows the scatter plots for all relevant relations.

**Figure 6:**
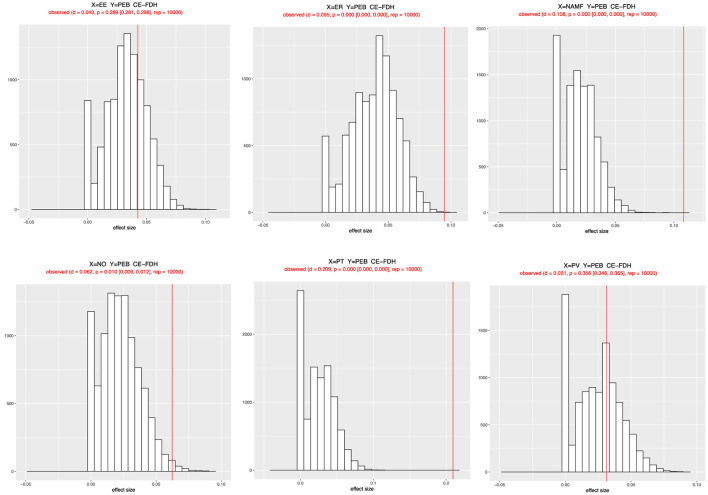
Histograms with bottleneck values.

We then used the CE-FDH line to avoid further linear assumptions, which is a non-decreasing step function derived from the scatterplot between the predictors and outcomes (Dul, 2016, 2021). In addition to having discrete data that covered a relatively narrow range and exhibited a limited number of levels, the CE-FDH ceiling line was justified (Dul, 2021). In addition, the parameters of the CE-FDH line from the NCA findings were employed for interpretation, as this line is acceptable for Likert-scale survey data (Vis and Dul, 2018). Thus, we were able to identify the extent to which each independent variable attribute limits overall pro-environmental behavior by separating the space containing observations from that containing no observations (Sukhov et al., 2022). Accordingly, the results in Table 6 and Figure 6 meaningfully (*d* ≥ 0.1) and significantly (*p* < 0.05) reveal that NAMF (*d* = 0.108, *p* < 0.001) and perceived threat (*d* = 0.209, *p* < 0.001) are necessary conditions for generating pro-environmental behavior. However, other variables had a small impact size and did not have a substantial influence on pro-environmental conduct (Dul, 2016).

**Table 6:** Necessary condition analysis result (method: CE-FDH).

**Construct**	**Ceiling zone**	**Scope**	**Effect size (d)**	***p* values**	**Conditional inefficiency (%)**	**Outcome inefficiency (%)**
EE	0.659	1.360	0.043	0.289	81.381	75.006
ER	1.483	15.643	0.095	^***^	26.575	66.328
NAMF	1.912	17.631	0.108	^***^	49.988	71.033
NO	0.992	15.922	0.062	^**^	44.701	79.026
PT	3.782	18.066	0.209	^***^	50	40.648
PV	0.518	16.451	0.031	0.356	87.352	75.006

*0 < d <0.15 small effect size; 0.1 ≤ d <0.3 medium effect size; 0.3 ≤ d <0.5 large effect size; d ≥ 0.5 very large effect size*.

* *p < 0.05*,

** *p < 0.01, and*

*** *p < 0.001*.

Finally, bottleneck tables were used to examine the ceiling line from a unique viewpoint, demonstrating which criteria and what level must be satisfied to attain the desired output level. Specifically, the bottleneck table of the NCA (Table 7) presents the threshold (critical) values for NAMF, environmental empathy, environmental responsibility, nostalgia, perceived threat, and perceived vulnerability to achieve the desired level of pro-environmental behavior value. The level of perceived threat should not fall below 6.9% to maintain a medium level of pro-environmental behavior (>40%). Similarly, to achieve a high level of pro-environmental behavior (80%), at least 5.6% of environmental empathy, 16.5% of environmental responsibility, 12.1% of NAMF, 39.6% of perceived threat, and 4.4% of perceived vulnerability must occur.

**Table 7:** Bottleneck Table (CE-FDH).

**PEB**	**EE**	**ER**	**NAMF**	**NO**	**PT**	**PV**
0	NN	NN	NN	NN	NN	NN
10	NN	NN	NN	NN	NN	NN
20	NN	NN	NN	NN	NN	NN
30	NN	NN	NN	NN	NN	NN
40	NN	NN	NN	NN	6.9	NN
50	NN	NN	NN	NN	15.1	NN
60	NN	NN	NN	NN	23.3	NN
70	NN	NN	NN	NN	31.4	NN
80	5.6	16.5	12.1	NN	39.6	4.4
90	14.1	41.9	30.8	20.6	47.8	12.8
100	22.6	67.3	49.4	50.6	56	21.2

## Discussion

### Theoretical contributions

This study integrated protection motivation theory, the SOR framework, and message framing theory at the same time, combining NAMF, environmental empathy, environmental responsibility, perceived threat, perceived vulnerability, and nostalgia into the model to evaluate how these variables affect pro-environmental behavior. The previous classification of anthropomorphic research was mainly based on the differentiation of social relationships (Karpinska-Krakowiak et al., 2020). This study integrated the framing effect and anthropomorphism, distinguished between positive and negative anthropomorphic message frames, and discussed them from the perspective of a negative message frame, enriching the theory of environmental behavior research and broadening its application. It is vital to understand people's behavioral mechanisms to motivate them to make sustainable decisions. Moreover, most previous studies used structural equation modeling and ordinary least squares regression techniques to analyze the factors that influence pro-environmental behavior. We used both sufficiency and necessity logic in research on pro-environmental behavior. PLS-SEM and NCA analyses revealed that the predictors of pro-environmental behavior were statistically significant. In addition, each predictor had varying degrees of necessity and net effects.

### Discussion of results

According to the PLS-SEM results, NAMF had a positive effect on environmental empathy and pro-environmental behavior. These results are in line with previous research (Tam et al., 2013; Chang and Wu, 2015; Moon et al., 2016; Amatulli et al., 2019; Liu et al., 2019; Tam, 2019; Karpinska-Krakowiak et al., 2020; Williams et al., 2021a; Borovik and Pensini, 2022). When anthropomorphism is coupled with negative information, empathy is triggered (Slovic, 2010) and researchers have found that when negative information is paired with anthropomorphic cues, they are more effective in inspiring green intentions (Karpinska-Krakowiak et al., 2020). Additionally, it was significantly associated with perceived threats and vulnerability. Interestingly, of the five direct relationships examined, NAMF was the strongest significant predictor of pro-environmental behavior. Consumers who receive negative anthropomorphic stimuli feel distressed and sympathetic toward the environment. They are usually in a negative mood, making them more sensitive to environmental issues and more determined to take steps to improve them. In addition, empathy for the environment leads to greater environmental responsibility, consistent with previous studies (Schultz P. W., 2000; Berenguer, 2007; Luchs and Mooradian, 2012; Stevenson et al., 2014). Higher levels of connection with nature are associated with increased prosocial behaviors and more sympathetic care for the biosphere and its inhabitants (Shelton and Rogers, 1981; Berenguer, 2007). Increased emotional empathy for nature increases a person's likelihood of acting properly; as a result, their love for nature is likely to grow (Boeve-de Pauw and Van Petegem, 2011). Empathy for nature is seen as a precondition for environmentally responsible behavior and has been linked to good environmental attitudes and knowledge (Chawla, 1998). Moreover, according to previous research, empathy is malleable and can be cultivated for future generations, which can lead to a variety of pro-environmental outcomes, such as greater engagement with environmental issues and pro-environmental behavioral intentions (Batson et al., 2003). Furthermore, perceived threat and perceived vulnerability were found to have a significant relationship with environmental empathy and environmental responsibility, respectively, relationships that have not been explored. In addition, perceived threat has a substantial influence on pro-environmental behavior, which is consistent with earlier results demonstrating the relationship between people's views of the threat posed by climate change and their desire to participate in pro-environmental activities (Grothmann and Patt, 2005; Spence et al., 2011; Bockarjova and Steg, 2014; Rainear and Christensen, 2017; Lim and Moon, 2020). Unexpectedly, the relationship between perceived vulnerability and pro-environmental factors was not significant, which is inconsistent with previous studies (Keshavarz and Karami, 2016; Bubeck et al., 2018) but in line with the research of Shafiei and Maleksaeidi (2020). In other words, higher perceived vulnerability did not affect individuals' perceptions of pro-environmental behaviors in this study. Not surprisingly, environmental responsibility positively influenced pro-environmental behavior, corroborating previous findings (Kaiser and Shimoda, 1999; Dermody et al., 2018; Han et al., 2020; Janmaimool and Khajohnmanee, 2020; Ahmed et al., 2021). Because environmental behavior benefits nature, other people, and society, it is likely to entail some cost to the actor, such as time, money, or energy. When a person is faced with a choice between personal and social interests, the internalized sense of environmental responsibility will be reawakened, leading the individual to engage in environmental behavior. We believe that consumers with a high sense of environmental responsibility will recognize their relationship with the environment as a duty, consider their obligation to solve environmental problems, and be more likely to adopt pro-environmental behaviors than consumers with a low sense of environmental responsibility. Finally, nostalgia was found to be a significant moderator and predictor of pro-environmental behavior. This implies that nostalgia augments the effects of environmental responsibility and negative anthropomorphic messages on pro-environmental behavior. It appears that nostalgia motivates pro-environmentalists (Rees et al., 2015). In general, the results of this study are consistent with those found in the literature regarding the effects of nostalgia on pro-environmental behavior. For example, Wu et al. (2020) added nostalgia to the framework of the TPB and found that nostalgia had a significant effect on pro-environmental behaviors through meaning in life and other core variables. Similarly, Zhang et al. (2021) focused on recycling behavior, which is a type of pro-environmental behavior, and found that nostalgia enhanced the intention to perform more environmentally friendly activities.

The NCA results revealed that NAMF (*d* = 0.108, *p* < 0.001) and perceived threat (*d* = 0.209, *p* < 0.001) were key factors of pro-environmental behavior. In addition, to receive a high level of pro-environmental behavior (>80%), NAMF (12.1%) and perceived threat (39.6%) are required, which means that marketers will not receive additional benefits above this level. Overall, incorporating the results of both PLS-SEM and NCA, we can conclude that NAMF and perceived threat are both necessary factors of pro-environmental behavior. Overall, the combined use of PLS-SEM and NCA provided more comprehensive knowledge of the sufficient and required antecedent factors for pro-environmental behavior.

### Managerial implications

The results of our study had several managerial implications: first, differentiated marketing strategies should be implemented for marketers. The findings of this study confirm the significant moderating effects of nostalgia on the relational structures of negative anthropomorphic message frames, perceived threats, and pro-environmental behavior, which marketers can use to develop differentiated management decisions. Individuals with a strong sense of nostalgia are more likely to trigger strong pro-environmental behaviors in their scheduled life when they are at the same level of environmental responsibility or perceive the same level of negative anthropomorphic external stimuli, whereas individuals with low levels of nostalgia have the exact opposite. Nostalgia marketing is a popular marketing method that uses nostalgic elements to encourage people to actively engage in pro-environmental behaviors; it is also a marketing method that is currently popular and well received by consumers. Therefore, marketing and services need to be targeted toward the masses with different nostalgic tendencies. For instance, marketers can identify individuals with low nostalgic tendencies and create a comprehensive nostalgic atmosphere by appealing to visual, auditory, and olfactory senses through social media green advertising and other methods to trigger individuals' nostalgic emotions through different sensory stimulations to further enhance their nostalgic memories. While promoting the pro-environmental behavior of individuals with a strong nostalgia tendency, we can increase nostalgia propaganda and dedicate more high-quality green nostalgia marketing content through precision social media marketing.

In addition, consumers should be aware of their environmental responsibilities. The government can use media, such as television and the internet, to disseminate general environmental knowledge to consumers and provide environmental training. This will enable them to understand the dichotomy between humans and the environment and trigger their emotional response to environmental issues. Furthermore, the government can also focus on environmental issues that directly affect consumers, such as water pollution, haze, and inform on them in a variety of ways, to make everyone aware that environmental issues need to be addressed, thus enhancing consumers' sense of urgency to protect the environment and inspiring a sense of responsibility. Finally, some consumers are not as informed or concerned about the environment as others (Kronrod et al., 2012). Therefore, persuading consumers to adopt environmentally friendly behaviors is never easy and often requires a high investment of time and money with modest returns. In the future, governments and marketers may combine negative message framing with anthropomorphic messages in green advertising or marketing campaigns, especially in China. Confucian culture heavily influences Chinese values and behavioral patterns and China is a country with a high-power distance where everyone subconsciously seeks their place within society. Centralistic governance and values have led Chinese consumers to attribute environmental problems to the government (De Silva et al., 2021). Thus, anthropomorphing nature can be empathetic and negative message framing makes consumers feel that what they are doing is important and urgent, which would greatly enhance the persuasive power of green advertising and marketing campaigns.

### Limitations and future research directions

Although this study contributed to the theoretical, methodological, and managerial domains, it had several limitations. First, as part of the research, we divided the message frames into positive and negative aspects; however, to focus this study, we only utilized negative message frames with anthropomorphism and examined the mechanisms that influenced pro-environmental behavior. Future research should focus on investigating the similarities and differences between negative and positive anthropomorphic message frames and whether positive anthropomorphism also evokes empathy for nature, environmental responsibility, perceived threat, and perceived vulnerability, which in turn affects consumers' pro-environmental behavior.

Second, we used the survey method, self-reported data, and a correlational design with inherent limitations that impose measurement error issues, social desirability bias, and a lack of causal conditions (Sharma et al., 2022a). Multiple research approaches should be combined in future studies to produce highly robust results. In addition, the research conclusion is one-sided as it only examines a sample of Chinese consumers and does not compare and analyze the different characteristics of different consumer groups in the process of green consumption under multicultural conditions. Future research should explore the path that motivates pro-environmental behavior by including samples from different cultural groups.

## Data availability statement

The raw data supporting the conclusions of this article will be made available by the authors, without undue reservation.

## Author contributions

SZ: conceptualization, methodology, data curation, writing—original draft, figure production, writing—review, and editing. YW: figure production, writing—review, and editing. All authors contributed to the article and approved the submitted version.

## Conflict of interest

The authors declare that the research was conducted in the absence of any commercial or financial relationships that could be construed as a potential conflict of interest.

## Publisher's note

All claims expressed in this article are solely those of the authors and do not necessarily represent those of their affiliated organizations, or those of the publisher, the editors and the reviewers. Any product that may be evaluated in this article, or claim that may be made by its manufacturer, is not guaranteed or endorsed by the publisher.
