# Supplementary material for: How negative anthropomorphic message framing and nostalgia enhance pro-environmental behaviors during the COVID-19 pandemic in China: An SEM-NCA approach
Source: Front Psychol. 2022 Aug 22;13:977381. doi: 10.3389/fpsyg.2022.977381 (PMC9441880; doi:10.3389/fpsyg.2022.977381)
Supplement: Supplementary file 1 [file Table_1.DOCX]

Appendix

Appendix 1 Cross loadings

|  | **EE** | **ER** | **NAMF** | **NO** | **PEB** | **PT** | **PV** |
| --- | --- | --- | --- | --- | --- | --- | --- |
| EE1 | **0.884** |  | 0.476 | 0.135 | 0.322 | 0.541 | 0.454 |
| EE2 | **0.878** | 0.446 | 0.426 | 0.076 | 0.341 | 0.447 | 0.274 |
| EE3 | **0.889** | 0.452 | 0.443 | 0.076 | 0.306 | 0.473 | 0.319 |
| EE4 | **0.748** | 0.329 | 0.357 | -0.032 | 0.235 | 0.367 | 0.204 |
| ER1 | 0.544 | **0.917** | 0.343 | 0.052 | 0.459 | 0.418 | 0.403 |
| ER2 | 0.398 | **0.829** | 0.368 | 0.160 | 0.413 | 0.377 | 0.375 |
| ER3 | 0.432 | **0.822** | 0.282 | 0.028 | 0.322 | 0.388 | 0.392 |
| NAMF1 | 0.535 | 0.377 | **0.865** | 0.231 | 0.555 | 0.513 | 0.355 |
| NAMF2 | 0.339 | 0.317 | **0.820** | 0.208 | 0.530 | 0.500 | 0.349 |
| NAMF3 | 0.327 | 0.275 | **0.743** | 0.209 | 0.471 | 0.399 | 0.273 |
| NAMF4 | 0.360 | 0.224 | **0.707** | 0.116 | 0.347 | 0.390 | 0.199 |
| NO1 | -0.013 | -0.027 | 0.045 | **0.715** | 0.094 | -0.076 | -0.092 |
| NO2 | 0.010 | -0.057 | 0.105 | **0.753** | 0.106 | 0.052 | -0.078 |
| NO3 | 0.140 | 0.136 | 0.261 | **0.899** | 0.283 | 0.164 | 0.013 |
| NO4 | 0.105 | 0.108 | 0.258 | **0.881** | 0.282 | 0.125 | -0.004 |
| NO5 | 0.006 | 0.071 | 0.193 | **0.802** | 0.212 | 0.070 | -0.035 |
| PEB1 | 0.343 | 0.447 | 0.508 | 0.212 | **0.827** | 0.494 | 0.149 |
| PEB2 | 0.270 | 0.368 | 0.507 | 0.272 | **0.823** | 0.464 | 0.197 |
| PEB3 | 0.267 | 0.361 | 0.442 | 0.169 | **0.743** | 0.410 | 0.210 |
| PEB4 | 0.254 | 0.339 | 0.492 | 0.185 | **0.804** | 0.422 | 0.189 |
| PEB5 | 0.247 | 0.347 | 0.471 | 0.172 | **0.783** | 0.417 | 0.189 |
| PEB6 | 0.306 | 0.359 | 0.501 | 0.283 | **0.780** | 0.509 | 0.189 |
| PT1 | 0.540 | 0.416 | 0.552 | 0.074 | 0.483 | **0.879** | 0.267 |
| PT2 | 0.426 | 0.387 | 0.467 | 0.150 | 0.447 | **0.813** | 0.259 |
| PT3 | 0.414 | 0.368 | 0.449 | 0.077 | 0.532 | **0.855** | 0.257 |
| PV1 | 0.341 | 0.404 | 0.361 | -0.019 | 0.217 | 0.272 | **0.871** |
| PV2 | 0.336 | 0.339 | 0.322 | -0.046 | 0.204 | 0.292 | **0.860** |
| PV3 | 0.319 | 0.404 | 0.313 | -0.014 | 0.209 | 0.221 | **0.864** |
| PV4 | 0.327 | 0.424 | 0.325 | -0.015 | 0.181 | 0.280 | **0.867** |

Note. NAMF-negative anthropomorphic message framing; PV-perceived vulnerability; PT-perceived threat; EE-environmental empathy EE; ER-environmental responsibility; PEB-pro environmental behavior; NO-nostalgia

**Appendix 2** Measurement Items Data Description

|  | Mean | Median | Standard Deviation | Excess Kurtosis | Skewness |
| --- | --- | --- | --- | --- | --- |
| NAMF1 | 2.453 | 2 | 1.203 | -0.912 | 0.385 |
| NAMF2 | 2.671 | 3 | 1.246 | -1.114 | 0.069 |
| NAMF3 | 2.645 | 2 | 1.27 | -1.035 | 0.304 |
| NAMF4 | 2.892 | 3 | 1.115 | -0.758 | -0.026 |
| EE1 | 2.761 | 2 | 1.349 | -1.119 | 0.384 |
| EE2 | 2.724 | 3 | 1.196 | -0.66 | 0.463 |
| EE3 | 2.897 | 3 | 1.276 | -1.022 | 0.422 |
| EE4 | 2.618 | 2 | 1.256 | -0.654 | 0.526 |
| PV1 | 2.874 | 3 | 1.131 | -0.689 | 0.097 |
| PV2 | 2.863 | 3 | 1.219 | -0.869 | 0.237 |
| PV3 | 2.645 | 2 | 1.222 | -0.756 | 0.488 |
| PV4 | 2.695 | 3 | 1.188 | -0.677 | 0.4 |
| PEB1 | 2.476 | 2 | 1.299 | -0.875 | 0.519 |
| PEB2 | 2.492 | 2 | 1.276 | -0.974 | 0.391 |
| PEB3 | 2.866 | 3 | 1.115 | -0.59 | 0.118 |
| PEB4 | 2.779 | 3 | 1.142 | -0.729 | 0.271 |
| PEB5 | 2.808 | 3 | 1.141 | -0.716 | 0.254 |
| PEB6 | 2.476 | 2 | 1.166 | -0.854 | 0.352 |
| ER1 | 2.679 | 3 | 1.234 | -0.699 | 0.41 |
| ER2 | 2.455 | 2 | 1.263 | -0.618 | 0.644 |
| ER3 | 3.013 | 3 | 1.282 | -1.147 | 0.118 |
| NO1 | 3.087 | 3 | 1.108 | -0.796 | 0.038 |
| NO2 | 3.026 | 3 | 1.187 | -0.909 | 0.015 |
| NO3 | 2.882 | 3 | 1.285 | -1.068 | 0.17 |
| NO4 | 2.624 | 2 | 1.317 | -1.012 | 0.351 |
| NO5 | 2.808 | 3 | 1.299 | -1.056 | 0.136 |
| PT1 | 2.963 | 3 | 1.13 | -0.718 | -0.081 |
| PT2 | 2.803 | 3 | 1.093 | -0.586 | 0.204 |
| PT3 | 2.905 | 3 | 1.077 | -0.606 | 0.037 |

**Appendix 3** Assessment of Structural Model (Control variable added)

| Hypothesis and Path | Coefficient | Std | T values | P Values |
| --- | --- | --- | --- | --- |
| Age -> PEB | 0.003 | 0.036 | 0.089 | 0.929 |
| Edu -> PEB | -0.048 | 0.035 | 1.382 | 0.167 |
| Gender -> PEB | 0.038 | 0.036 | 1.052 | 0.293 |
| Income -> PEB | 0.008 | 0.037 | 0.221 | 0.825 |
| EE -> ER | 0.328 | 0.049 | 6.625 | *** |
| ER -> PEB | 0.207 | 0.053 | 3.879 | *** |
| NAMF -> EE | 0.227 | 0.052 | 4.375 | *** |
| NAMF -> PEB | 0.304 | 0.057 | 5.297 | *** |
| NAMF -> PT | 0.578 | 0.037 | 15.839 | *** |
| NAMF -> PV | 0.382 | 0.047 | 8.178 | *** |
| NO -> PEB | 0.117 | 0.037 | 3.173 | 0.002 |
| PT -> EE | 0.356 | 0.047 | 7.553 | *** |
| PT -> ER | 0.199 | 0.053 | 3.788 | *** |
| PT -> PEB | 0.257 | 0.052 | 4.908 | *** |
| PV -> EE | 0.186 | 0.047 | 3.909 | *** |
| PV -> ER | 0.268 | 0.046 | 5.779 | *** |
| PV -> PEB | -0.064 | 0.043 | 1.483 | 0.138 |
| PV*NO -> PEB | -0.007 | 0.044 | 0.159 | 0.873 |
| PT*NO -> PEB | -0.03 | 0.045 | 0.674 | 0.500 |
| NAMF*NO -> PEB | 0.146 | 0.058 | 2.511 | 0.012 |
| ER*NO -> PEB | 0.13 | 0.053 | 2.443 | 0.015 |

Appendix 4 Construct items

| Construct | Coding | Item |
| --- | --- | --- |
| Negative anthropomorphic message framing | NAMF1 | The image looks similar to a human being |
|  | NAMF2 | The image has a human-like mind |
|  | NAMF3 | The image has some emotion |
|  | NAMF4 | The image can communicate like a human being |
| PT | PT1 | I will be threatened by the environment |
|  | PT2 | I feel in great danger |
|  | PT3 | I feel that I will suffer a loss |
| PV | PV1 | I feel I have a high likelihood of being affected by environmental damage |
|  | PV2 | I am vulnerable to loss from environmental damage |
|  | PV3 | I will be a victim of environmental damage if certain measures are not taken |
|  | PV4 | I will experience the negative effects of environmental degradation in my lifetime |
| PEB | PEB1 | Would you like to separate newspapers, plastic and glass bottles? |
|  | PEB2 | Would you like to reduce your driving to protect the environment? |
|  | PEB3 | Would you like to recycle water to protect the environment? |
|  | PEB4 | Would you like to participate in environmental activities organized by charity organizations? |
|  | PEB5 | Would you like to buy organic vegetables and fruits? |
|  | PEB6 | Would you like to make a donation to an environmental organization? |
| Nostalgia | NO1 | Thinking about this event puts me in a good mood |
|  | NO2 | Thinking about this event makes me value myself more |
|  | NO3 | Thinking about this event makes me feel connected to loved ones |
|  | NO4 | Thinking about this event makes me feel that life is worth living |
|  | NO5 | Thinking about this event makes me feel life is meaningful |
| EE | EE1 | I can immerse myself in the feelings of the earth |
|  | EE2 | I can easily put myself in the Earth's situation |
|  | EE3 | I have a clear picture of the Earth's plight in my mind |
|  | EE4 | I can feel the pain that the Earth is suffering |
| ER | ER1 | I have a responsibility to do my part to conserve resources and protect the environment |
|  | ER2 | I will take the initiative to learn about environmental protection |
|  | ER3 | I want to contribute to environmental protection, even though my impact is small |

Appendix 5 *Manipulation Checks material (pictures)* of negative framing

| 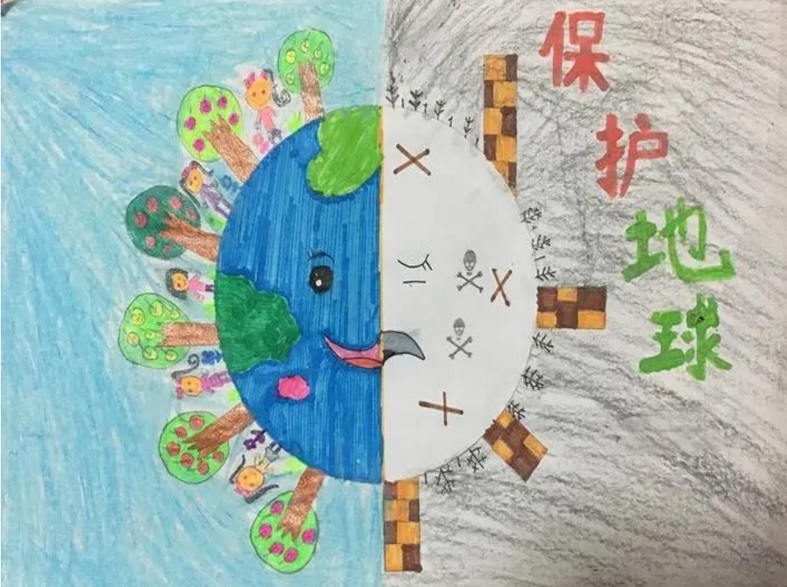 | 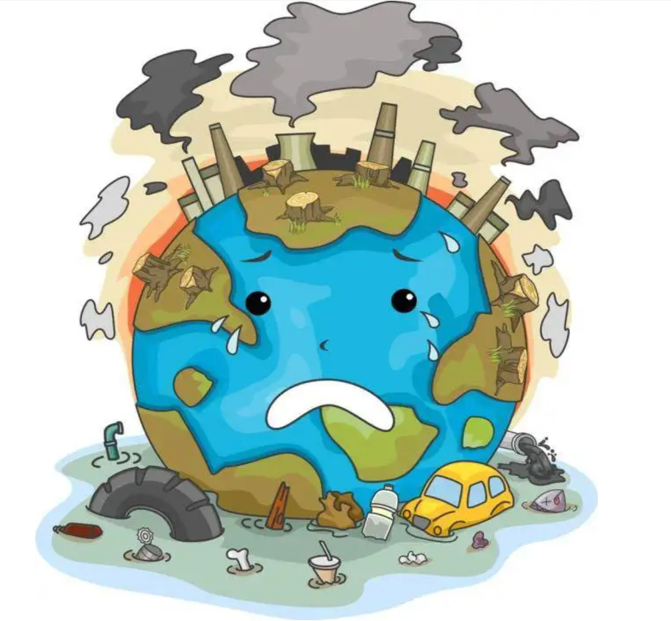 |
| --- | --- |

**Appendix 6** Results of the inner model (control variable added)


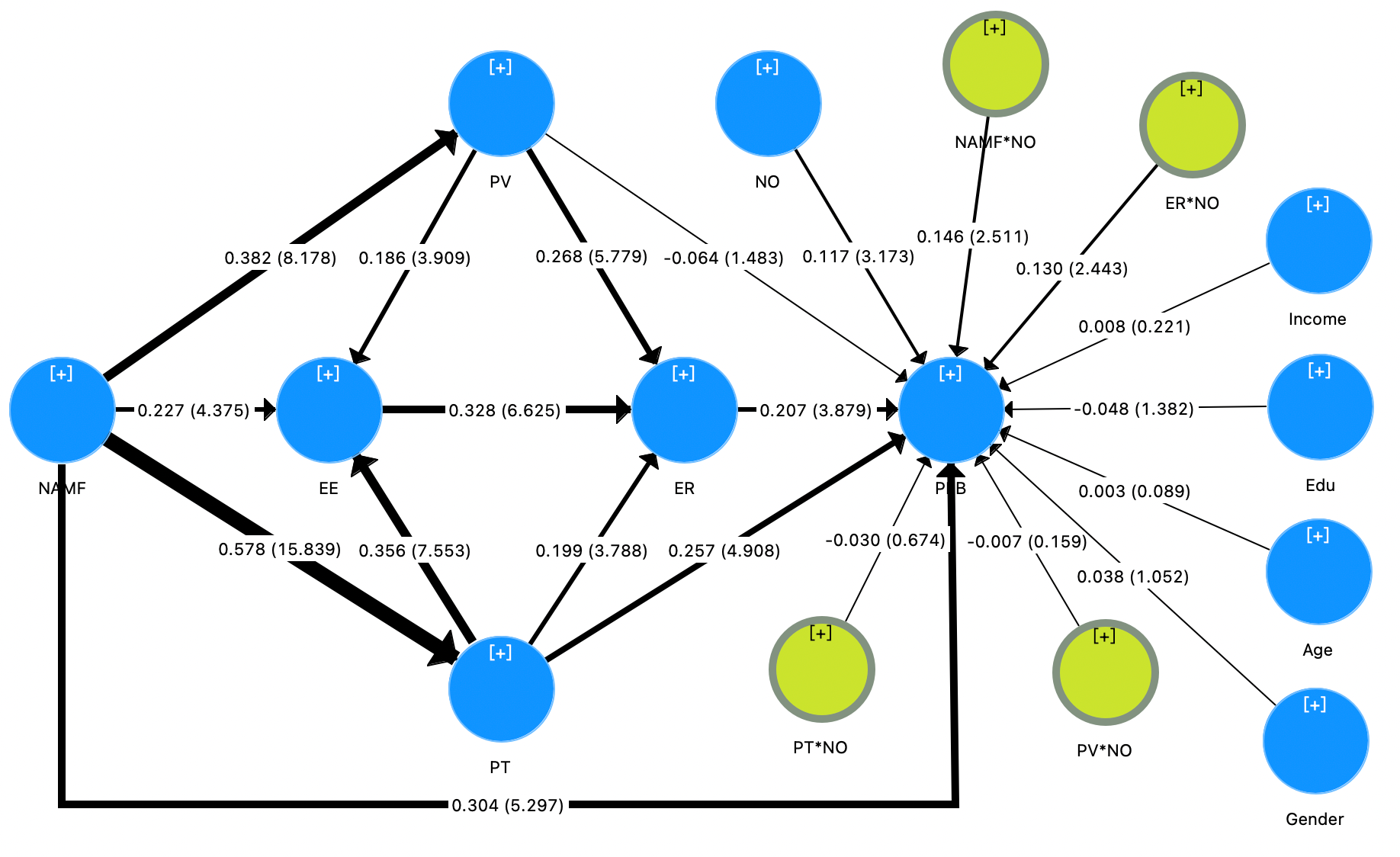


**Appendix 7** Non-response bias test

| Variable | T-test for  Equality of Means | df | Sig.  (2-tailed) | Mean  Difference | Std.  Error | 95% Confidence  Interval of the Difference | |
| --- | --- | --- | --- | --- | --- | --- | --- |
|  |  |  |  |  |  | Lower | Upper |
| Gender | -0.902 | 378 | **0.368** | -0.046 | 0.052 | -0.148 | 0.055 |
| Age | -0.023 | 378 | **0.981** | -0.002 | 0.101 | -0.200 | 0.196 |
| Edu | 1.919 | 378 | **0.056** | 0.158 | 0.082 | -0.004 | 0.320 |
| Income | -0.549 | 378 | **0.583** | -0.063 | 0.115 | -0.290 | 0.164 |
| NAMF | -0.236 | 378 | **0.813** | -0.023 | 0.098 | -0.216 | 0.170 |
| EE | 0.451 | 378 | **0.652** | 0.050 | 0.112 | -0.169 | 0.270 |
| PV | 0.609 | 378 | **0.543** | 0.065 | 0.106 | -0.144 | 0.274 |
| PEB | 0.995 | 378 | **0.321** | 0.097 | 0.097 | -0.095 | 0.289 |
| ER | 0.474 | 378 | **0.636** | 0.053 | 0.111 | -0.166 | 0.271 |
| NO | 0.343 | 378 | **0.732** | 0.036 | 0.105 | -0.170 | 0.243 |
| PT | 0.004 | 378 | **0.997** | 0.000 | 0.096 | -0.189 | 0.190 |
